# Granulocyte–macrophage colony-stimulating factor expression in induced sputum and bronchial mucosa in asthma and COPD

**DOI:** 10.1136/thx.2008.108290

**Published:** 2009-02-12

**Authors:** S Saha, C Doe, V Mistry, S Siddiqui, D Parker, M Sleeman, E S Cohen, C E Brightling

**Affiliations:** 1 Institute of Lung Health, Leicester, UK; 2 MedImmune Ltd, Milstein Building, Granta Park, Cambridge, UK

## Abstract

**Background:**

Granulocyte–macrophage colony-stimulating factor (GM-CSF) has been implicated as an important mediator in the pathogenesis of asthma and chronic obstructive pulmonary disease (COPD). However, the expression of GM-CSF and its receptor in airway samples in asthma and COPD across disease severity needs to be further defined.

**Methods:**

Sputum GM-CSF was measured in 18 control subjects, 45 subjects with asthma and 47 subjects with COPD. Enumeration of GM-CSF+ cells in the bronchial submucosa and airway smooth muscle bundle was performed in 29 control subjects, 36 subjects with asthma and 10 subjects with COPD.

**Results:**

The proportion of subjects with measurable GM-CSF in the sputum was raised in those with moderate (7/14) and severe (11/18) asthma, and in those with COPD GOLD (Global Initiative for Chronic Obstructive Lung Disease) stage II (7/16), III (8/17) and IV (7/14) compared with controls (1/18) and those with mild asthma (0/13); p = 0.001. The sputum GM-CSF concentration was correlated with the sputum eosinophilia in subjects with moderate to severe asthma (r_s_ = 0.41; p = 0.018). The median (interquartile range) GM-CSF+ and GM-CSFR+ cells/mm^2^ of submucosa was increased in severe asthma (1.4 (3.0) and 2.1 (8.4)) compared with those with mild to moderate asthma (0 (2.5) and 1.1 (5)) and healthy controls (0 (0.5) and 0 (1.6)), (p = 0.004 and p = 0.02, respectively).

**Conclusions:**

The findings support a potential role for GM-CSF in asthma and COPD and suggest that overexpression of GM-CSF in sputum and the bronchial mucosa is a particular feature of severe asthma.

The airway diseases asthma and chronic obstructive pulmonary disease (COPD) are common and cause significant morbidity and mortality worldwide. Asthma affects 10% of children and 5% of adults, and its prevalence continues to rise.^1^ Severe asthma accounts for about 10% of asthma, but is particularly important as it leads to debilitating chronic symptoms despite optimal standard asthma treatment and contributes to over half of the healthcare costs attributed to asthma.^1^^–^3 COPD is a major public health problem and will rank as the third cause of death in 2030.^4^ Both conditions are characterised by airflow obstruction with airway inflammation, and remodelling. Although the inflammatory profiles of asthma and COPD have been described as overlapping,^5^ asthma is more commonly associated with Th2-mediated eosinophilic inflammation^6^ whereas in COPD neutrophilic inflammation is more predominant.^5^ Several cytokines and chemokines have been implicated in driving the airway inflammatory response in asthma and COPD.

Granulocyte–macrophage colony-stimulating factor (GM-CSF) is a major regulator of inflammatory cells of the myeloid lineage and has been implicated in asthma and COPD.^7^ It is released by a range of structural and inflammatory cells, including airway epithelium, airway smooth muscle (ASM), fibroblasts, T lymphocytes, mast cells, eosinophils and macrophages. GM-CSF has recently been shown to signal via a ternary receptor complex (GM-CSFR) composed of a 2:2:2 hexamer consisting of two βc chains, two GMRα chains and two GM-CSF molecules.^8^ GM-CSF is a pleiotrophic and proinflammatory cytokine that stimulates myelopoiesis, promotes leucocyte survival and activation, and regulates mucosal immunity and inflammation in part via modulation of Toll-like receptor-4^9^ and neutrophil function.^10^ Its importance in airways disease is supported by evidence from mouse models of COPD^7^ and asthma,^11^ whereby administration of anti-GM-CSF antibody attenuates the neutrophilic and eosinophilic inflammatory response, respectively. Importantly, in human disease, GM-CSF expression is increased in sputum, bronchoalveolar lavage (BAL) and bronchial biopsies in asthma.^12^^–^17 In contrast, in COPD there is a lack of direct evidence of increased GM-CSF expression in airway secretions or biopsy tissue. However, in culture, GM-CSF secretion by ex vivo sputum cells is increased in COPD.^18^ Similarly, whether GM-CSFR expression is increased in airways disease is contentious, with one study suggesting that GM-CSFR is increased in non-atopic, but not atopic asthma.^19^ Therefore, GM-CSF and GM-CSFR expression in airways disease needs to be further defined.

We hypothesised that GM-CSF and GM-CSFR expression is increased in asthma and COPD, and is related to disease severity. To test our hypothesis we have measured the sputum GM-CSF concentration and enumerated in bronchial mucosa the number of GM-CSF+ and GM-CSFR+ cells in asthma and COPD.

## METHODS

### Subjects

Subjects were recruited from hospital staff, the general respiratory and the ‘Difficult Asthma’ clinics at Glenfield Hospital, Leicester, local primary healthcare and by local advertising. Asthma was defined according to the current Global Initiative for Asthma (GINA) guidelines.^20^ Subjects with asthma had typical symptoms and the presence of one or more of the following objective criteria: significant bronchodilator reversibility of forced epiratory volume in 1 s (FEV_1_) >200 ml, a provocation concentration of methacholine causing a 20% fall in FEV_1_ (PC_20_) of <8 mg/ml or a peak flow amplitude percentage mean over 2 weeks of >20%. Asthma severity was classified using the GINA treatment steps.^20^ COPD was diagnosed and severity categorised by using the Global Initiative for Chronic Obstructive Lung Disease (GOLD) criteria.^21^ Subjects with COPD who demonstrated partial bronchodilator reversibility were not excluded. Subjects were recruited as three independent cross-sectional groups, to assess sputum GM-CSF concentration in asthma and COPD (group 1); and GM-CSF and GM-CSFR expression in proximal airways in asthma (group 2) and COPD (group 3). Subjects were free from exacerbations for at least 6 weeks. Healthy controls had normal spirometry, and some smokers with >10 pack-year history were included to enable comparisons between healthy smokers and COPD subjects. All subjects gave written informed consent, with study approval from the Leicestershire ethics committee.

### Protocol

For all subjects, demographics and spirometry were recorded. Subjects with asthma and healthy controls in groups 1 and 2 also underwent a methacholine inhalation test using the tidal breathing method^22^ and allergen skin prick tests for *Dermatophagoides pteronyssinus*, dog, cat and grass pollen. Sputum induction using incremental concentrations of nebulised hypertonic saline 3, 4 and 5% each for 5 min was also performed in all subjects in groups 1 and 2.^23^

In group 2, subjects underwent bronchoscopy conducted according to the British Thoracic Society guidelines,^24^ and biopsies were taken from the right middle and lower lobe carinae. In group 3, proximal airway samples were collected from surgical specimens. All bronchial mucosal specimens were fixed in acetone and embedded in glycomethacrylate as described previously.^25^

### Sputum GM-CSF measurement

Sputum was selected, dispersed using the mucolytic dithiothreitol (DTT) and processed to generate a sputum differential cell count, and cell-free supernatants were stored at −80°C for later analysis as described previously.^26^

Sputum GM-CSF was measured by ELISA (Caltag-Medsystems, Buckinghamshire, UK). The lower limit of detection was 10 pg/g sputum. The GM-CSF assay was validated in line with European Respiratory Society recommendations to assess the effect of DTT and the recovery of exogenous spiking with recombinant cytokine.^27^ GM-CSF recovery was not affected by DTT, and recovery of exogenous spiked GM-CSF was 103% (15%) (n = 4).

### GM-CSF and GM-CSFR expression in endobronchial biopsies

Sections of 2 μm were cut and stained using monoclonal antibodies against GM-CSF (clone: BVD2-21C11, Cambridge BioScience, Cambridge, UK), GM-CSFR (clone: 2B7, a monoclonal antibody raised to the extracellular domain of GM-CSFR, gift from Dr Sleeman. MedImmune, Grant Park, Cambridge, UK), or appropriate isotype controls (rat immunoglobulin G2a (IgG2a) (R&D Systems Europe, Abingdon, UK) and mouse IgG1 clone DAK-Go1 (Dako UK, Cambridge, UK), respectively). The number of positive nucleated cells was enumerated per mm^2^ of bronchial submucosa or ASM bundle by a blinded observer as described previously.^28^ ^29^

### Statistical analysis

Statistical analysis was performed using PRISM Version 4. Parametric data were expressed as mean (SEM), data that had a normal log distribution were log transformed and described as geometric mean (95% CI) and non-parametric data were described as median (interquartile range (IQR)). One-way analysis of variance (Kruskal–Wallis for non-parametric data) was used for across-group comparisons, with Tukey and Dunn post hoc tests for between-group comparisons. χ^2^ tests were used to compare categorical data. Correlations were assessed by Spearman rank correlation coefficients.

## RESULTS

### Sputum GM-CSF concentration in asthma and COPD

**Figure 1:**
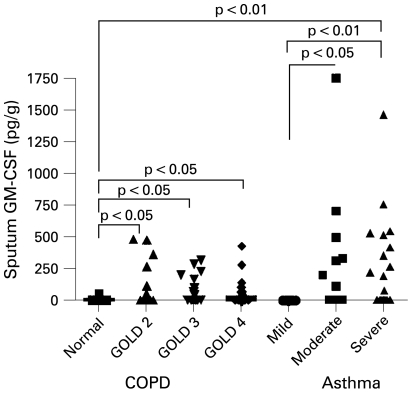
Sputum granulocyte–macrophage colony-stimulating factor (GM-CSF) concentration. Sputum GM-CSF concentration in the control group, subjects with chronic obstructive pulmonary disease (COPD) (Global Initiative for Chronic Obstructive Lung Disease (GOLD) II–IV), mild asthma (Global Initiative for Asthma (GINA) 1), moderate asthma (GINA 2–4) or severe asthma (GINA 5). Across-group comparisons by Kruskal–Wallis test were p<0.05; p values for Dunn post hoc test for between-group comparisons are as shown.

Clinical and sputum characteristics for subjects in group 1 are shown in table 1. The proportion of subjects with measurable GM-CSF in the sputum was raised in those with moderate (7/14) and severe (11/18) asthma, and in those with COPD GOLD stage II (7/16), III (8/17) and IV (7/14) compared with controls (1/18) and those with mild asthma (0/13) (p = 0.001). The sputum GM-CSF concentration was increased in subjects with COPD across severity compared with controls (p = 0.02 Kruskal–Wallis; p<0.05 for COPD all severities compared with controls; fig 1). Similarly, the sputum GM-CSF concentration was increased in severe asthma compared with mild asthma and controls, and in moderate asthma compared with mild disease (p<0.001 Kruskal–Wallis; p<0.05 for between-group comparisons; fig 1). The sputum GM-CSF concentration was increased in the subjects with moderate and severe asthma combined compared with those with COPD GOLD II–IV combined (p = 0.004). The sputum GM-CSF concentration was correlated with the sputum eosinophilia in subjects with disease as a whole group (r_s_ = 0.28; p = 0.007), all those with asthma (r_s_ = 0.3; p = 0.04) and moderate and severe disease (r_s_ = 0.41; p = 0.018), but not COPD. There was no association in subjects with asthma or COPD with sputum GM-CSF concentration and percentage predicted FEV_1_ (r = −0.26, p = 0.09; r = −0.07, p = 0.7) or the FEV_1_/FVC (forced vital capacity) ratio (r = 0.06, p = 0.7; r = 0.1, p = 0.5), respectively.

**Table 1:** Group 1: clinical and sputum characteristics of subjects with asthma and chronic obstructive pulmonary disease

	Normal	Mild asthma (GINA 1)	Moderate asthma (GINA 2–4)	Severe asthma (GINA 5)	GOLD II	GOLD III	GOLD IV
Number	18	13	14	18	16	17	14
Age*	54 (3)	53 (4)	51 (4)	49 (5)	71 (2)	68 (2)	72 (11)
Male/female	4/14	8/5	4/10	7/11	9/7	12/5	13/1
Never/current/ex-smokers	9/0/9	11/0/11	10/0/4	16/0/2	2/9/5	0/6/11	0/4/10
Pack-years*	17 (5)	2 (1)	6 (3)	4 (3)	43 (8)	52 (4)	56 (9)
Atopy, n (%)	6 (33)	7 (54)	11 (79)	13 (72)	7 (44)	7 (41)	5 (36)
PC_20_FEV_1_ (mg/ml)†	>16	1 (0.3 to 4.2)	0.4 (0.1 to 1.5)	0.1 (0 to 1.6)	–	–	–
FEV_1_, % predicted*	98.7 (3.0)	80.4 (5.1)	66.4 (4.4)	56.4 (6.8)	60.1 (1.5)	39.9 (1.3)	24.1 (1.3)
Pre-BD FEV_1_/FVC, %*	77.6 (1.7)	72.1 (3.6)	67.5 (3.0)	69.7 (2.8)	59.4 (2.2)	50.9 (2.1)	40.3 (2.0)
BD response (%)*	1.5 (0.7)	6.0 (3.6)	5.0 (4.6)	9.6 (2.0)	4.3 (2.2)	6.0 (1.8)	4.1 (3.2)
Sputum cell counts							
TCC*	3.8 (0.9)	2.34 (0.82)	3.31 (1.8)	6.57 (3.3)	3.3 (0.6)	4.3 (0.9)	11.2 (3.2)
Eosinophils, %†	0.5 (0.3 to 0.8)	2.3 (0.6 to 7.9)	2.7 (1.0 to 7.3)	3.8 (1.8 to 8.0)	2.3 (1.7 to 6.3)	2.6 (1.4 to 4.7)	1.0 (0.4 to 2.3)
Neutrophils, %*	55.8 (6.0)	67.3 (6.9)	57.8 (5.9)	64.3 (6.0)	72.2 (5.0)	71.0 (4.3)	85.6 (2.9)
Macrophages, %*	38.5 (5.3)	20.6 (4.6)	21.4 (4.7)	19.2 (4.9)	29.2 (4.6)	21.3 (3.3)	8.7 (1.7)
Lymphocytes, %*	0.4 (1.4)	0.5 (0.2)	0.4 (0.1)	1.9 (1.5)	1.6 (1.1)	0.6 (0.2)	1.0 (0.3)
Epithelial cells, %*	3.7 (1.4)	3.7 (1.8)	3.6 (1.7)	3.3 (1.6)	3.3 (0.6)	4.3 (0.9)	11.2 (3.2)

*Mean (SE). †Geometric mean (95% CI).

BD, bronchodilator; FEV_1_, forced expiratory voume in 1 s; FVC, forced vital capacity; GINA, Global Initiative for Asthma; GOLD, Global Initiative for Chronic Obstructive Lung Disease; PC, provocation concentration; TCC, total cell count.

### GM-CSF/R expression in large airway tissue specimens

**Figure 2:**
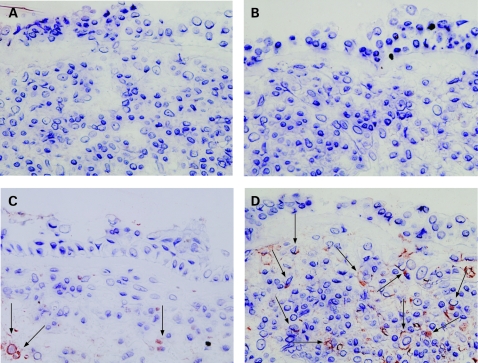
Examples of granulocyte–macrophage colony-stimulating factor-positive (GM-CSF+) and GM-CSF receptor-positive (GM-CSFR+) cells in the submucosa and airway smooth muscle bundle in asthma. Representative photomicrographs of bronchial biopsy sections from subjects with severe asthma illustrating isotype controls. (A) Rat immunoglobulin 2a (IgG2a), (B) mouse IgG1, (C) GM-CSF+ cells present in the bronchial submucosa and (D) GM-CSFR+ cells in the submucosa (×400). GM-CSF/R+ cells are highlighted by arrows.

**Figure 3:**
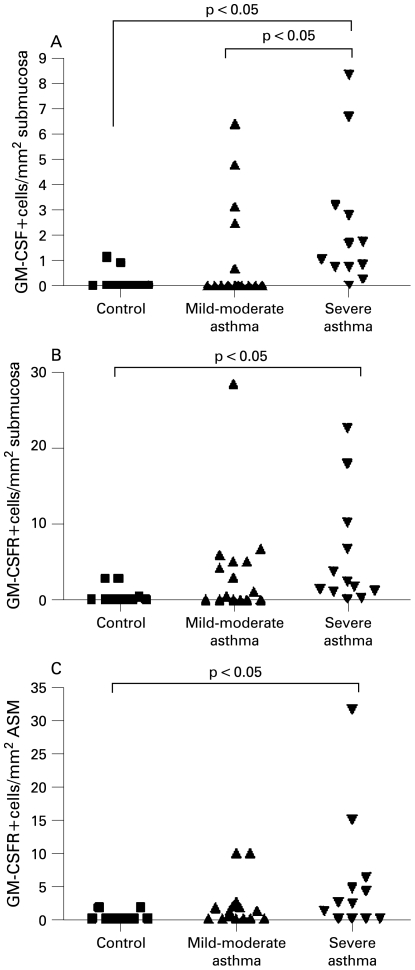
The number of granulocyte–macrophage colony-stimulating factor-positive (GM-CSF+) and GM-CSF receptor-positive (GM-CSFR+) cells in the submucosa in asthma. The number of (A) GM-CSF+ and (B) GM-CSFR+ cells in the bronchial submucosa and (C) GM-CSFR+ cells in the airway smooth muscle bundle in healthy controls, those with mild to moderate asthma (Global Initiative for Asthma (GINA) 1–3) and those with severe asthma (GINA 4–5). Across-group comparisons by Kruskal–Wallis test were all p<0.05; p values for Dunn post hoc test for between-group comparisons are as shown.

Examples of GM-CSF and GM-CSFR+ cells in the bronchial submucosa in asthma are as shown in fig 2. Clinical characteristics of group 2 are as shown in table 2. The median (IQR) GM-CSF cells/mm^2^ of submucosa was increased in severe asthma (1.4 (3.0)) compared with those with mild to moderate asthma (0 (2.5)) and healthy controls (0 (0.5)), (p = 0.004, Kruskal–Wallis; between-group comparisons are as shown fig 3A). The number of GM-CSFR+ cells/mm^2^ of submucosa and ASM was increased in severe asthma (2.1 (8.4) and 2.4 (5.5)) compared with healthy controls (0 (1.6) and 0 (0.8), but not in those with mild to moderate asthma (1.1 (5) and 1.2 (2.2)) (p = 0.02 and p = 0.049, respectively, Kruskal–Wallis; p<0.05 severe asthma vs control, fig 3B,C). The number of GM-CSF+ cells in the ASM bundle was very low in subjects with asthma and healthy controls.

**Table 2:** Group 2: clinical sputum characteristics of patients with asthma and healthy control subjects

	Normal	Mild to moderate asthma (GINA 1–3)	Severe asthma (GINA 4–5)
Number	10	15	12
Age*	38 (4)	48 (4)	50 (4)
Male/female	4/6	8/7	6/6
Never/current/ex-smokers	9/0/1	9/0/6	10/0/2
Pack years*	0 (0)	3 (1)	3 (2)
Atopy, (%)	5 (50)	10 (66)	9 (75)
PC_20_FEV_1_ (mg/ml)†	>16	0.3 (0.1 to	0.4 (0.1 to
FEV_1_, % predicted*	98.4 (4.5)	89.7 (4.9)	80.2 (6.6)
Pre-BD FEV_1_/FVC, %*	77.9 (3.3)	74.4 (2.9)	74.5 (3.4)
BD response (%)*	1.1 (1.2)	8.8 (4.1)	12.5 (5.7)
Sputum cell counts			
TCC*	2.1 (0.6)	2.9 (0.7)	2.7 (0.6)
Eosinophils, %†	0.4 (0.1 to 0.8)	0.9 (0.4 to 2.8)	2.9 (0.8 to 10.6)
Neutrophils, %*	48.8 (17.7)	55.2 (7.0)	59 (9.7)
Macrophages, %*	47.4 (11.7)	37.1 (6.3)	25.1 (5.8)
Lymphocytes, %*	1.9 (1.2)	1.1 (0.2)	1.5 (0.7)
Epithelial cells, %*	1.4 (1.2)	4.0 (1.6)	6.9 (3.3)
GM-CSF			
Submucosa‡	0 (0.5)	0 (2.5)	1.4 (3.0)
ASM‡	0 (0)	0 (0)	0 (0)
GM-CSFR			
Submucosa‡	0 (1.6)	1.1 (5)	2.1 (8.4)
ASM‡	0 (0.8)	1.2 (2.2)	2.4 (5.5)

*Mean (SE). †Geometric mean (95% CI). ‡Median (interquartile range).

ASM, airway smooth muscle; BD, bronchodilator; FEV_1_, forced expiratory voume in 1 s; FVC, forced vital capacity; GINA, Global Iniative for Asthma; GM-CSF, granulocyte–macrophage colony-stimulating factor; GM-CSFR, granulocyte–macrophage colony-stimulating factor receptor; PC, provocation concentration; TCC, total cell count.

There were no differences in the number of GM-CSF+ or GM-CSFR+ cells within the submucosa or ASM bundle in lung resection tissue from subjects with COPD and controls with and without a significant smoking history (table 3).

**Table 3:** Group 3: clinical characteristics and GM-CSF/GM-CSFR expression in proximal airway from lung resection

	Normal	Smoker	COPD
Number	8	11	10
Age*	58 (3)	60 (3)	66 (3)
Male/female	7/1	8/3	7/3
Never/current/ex-smokers	6/0/2	0/0/11	0/0/10
Pack-years*	0 (1)	30 (7)	39 (6)
FEV_1_*	2.8 (0.2)	2.6 (0.2)	1.8 (0.2)
FEV_1_, % predicted*	87 (3)	87 (4)	64 (4)
Pre-BD FEV_1_/FVC*	79 (3)	81 (4)	55 (3)
GM-CSF			
Submucosa†	0.5 (1)	0.8 (2.4)	0.2 (1.1)
ASM†	0 (0)	0 (0.1)	0 (0)
GM-CSFR			
Submucosa†	2.3 (6.1)	0.3 (1.3)	0.5 (4.4)
ASM†	0 (0.1)	0 (0)	0 (0)

*Mean (SE). †Median (interquartile range).

ASM, airway smooth muscle; BD, bronchodilator; COPD, chronic obstructive pulmonary disease; FEV_1_, forced expiratory voume in 1 s; FVC, forced vital capacity; GM-CSF, granulocyte–macrophage colony-stimulating factor; GM-CSFR, granulocyte–macrophage colony-stimulating factor receptor; PC, provocation concentration.

## DISCUSSION

We report here for the first time that the sputum GM-CSF concentration was increased in COPD, independent of disease severity, and confirm that in asthma the sputum GM-CSF concentration is associated with more severe disease. In asthma our sputum findings were supported by increased GM-CSF and GM-CSFR expression in bronchial biopsies in severe disease. Our study therefore supports our hypothesis that GM-CSF and GM-CSFR expression is increased in asthma and COPD, and in asthma is related to disease severity.

Several lines of evidence support a role for GM-CSF in COPD. GM-CSF is induced by the presence of airway pathogens^30^ ^31^ and is known to be an important regulator of the activation and survival of key effector cells in COPD, namely the neutrophil and macrophage.^32^ ^33^ Critically, neutralisation of GM-CSF in animal models attenuates airway inflammation in response to cigarette smoking.^7^ However, to date there has been a paucity of direct evidence of increased GM-CSF expression in airway secretions. Indeed the sputum GM-CSF concentration was not increased in subjects at exacerbations compared with their stable state at recovery,^34^ although in contrast GM-CSF release by sputum cells in culture was increased.^18^ In vivo and in vitro GM-CSF is rapidly internalised following receptor binding and therefore it is likely that the measurement of sputum GM-CSF is underestimated by ELISA.^7^ ^35^ Importantly, the concentration of free GM-CSF is under tight control, with measurable GM-CSF autoantibodies in healthy controls as well as in those with disease.^36^ In spite of this, we here have validated the measurement of GM-CSF in sputum and found that it was increased in subjects with COPD across all disease severities compared with smoking and non-smoking controls, although there was no relationship between sputum GM-CSF concentration and disease severity. In the resection samples, we were unable to confirm that expression of GM-CSF or its receptor was increased. However, we only studied subjects with COPD with milder disease, and the control subjects often had underlying lung cancer which may have masked differences between COPD and controls. Therefore, the role of GM-CSF in COPD needs to be further defined, and future studies need to include analysis of bronchial tissue in moderate to severe COPD.

In asthma there is a wealth of data supporting a role for GM-CSF. In particular GM-CSF is pivotal in eosinophil maturation and survival,^37^ a key effector cell in asthma. In animal models GM-CSF neutralisation attenuates airway inflammation, and GM-CSF knockout mice^38^ do not develop a bronchial eosinophilia in response to allergen challenge. In contrast to COPD, in asthma there are several reports of increased GM-CSF expression in airway secretions and tissue.^12^^–^17 In particular, increased sputum GM-CSF expression is associated with more severe disease.^12^ ^13^ We have confirmed these earlier reports and found that sputum GM-CSF concentration was increased in moderate to severe asthma, but not in mild disease. In addition the intensity of the sputum GM-CSF expression was related to the sputum eosinophilia. We report here for the first time that GM-CSF and GM-CSFR expression was also increased in the bronchial submucosa in more severe asthma. Therefore, in severe asthma there is a generalised upregulation in the GM-CSF/GM-CSFR axis, suggesting that this mediator may play a prominent role in severe asthma.

Our study design allowed for direct comparison of the sputum GM-CSF concentration in asthma and COPD, but not expression in tissue as samples were obtained using different methods. Comparisons in sputum GM-CSF concentrations were undermined by the relative insensitivity of our assay, with a large number of subjects that had concentrations below the level of detection of our assay. This is likely to reflect the rapid internalisation of GM-CSF. In spite of this limitation we found that sputum GM-CSF was increased in both COPD and moderate to severe asthma, and importantly it was greater in moderate to severe asthma than in COPD. Therefore, whether GM-CSF plays a more important role in severe asthma than in COPD warrants further investigation.

Our study has a number of possible criticisms. This is a cross-sectional observational study. We were unable to demonstrate an association between GM-CSF expression and lung function. Whether GM-CSF expression is related to longitudinal clinical outcomes such as disease progression, lung function decline and exacerbations requires further examination. Similarly, we are unable to determine whether differences observed between mild and severe asthma reflect disease severity or are a consequence of differences in treatment. Therefore, the effects of corticosteroids on GM-CSF need to be fully elucidated, although previous work suggests that GM-CSF expression in tissue is attenuated by corticosteroids.^39^ The rapid turnover of GM-CSF in vivo limits the interpretation of protein expression by ELISA and immunohistochemistry. We have not defined the cellular source of GM-CSF or determined whether the increased expression of GM-CSF is associated with an increase in the total number of infiltrating cells within the bronchial submucosa. In addition, protein expression in tissue often reflects granular stores and may underestimate GM-CSF expression in cells that release rather than store GM-CSF, such as T cells. Further studies are therefore required to confirm our findings and to determine the relative expression of GM-CSF in bronchial tissue by different cell types.

In conclusion, we found that the sputum GM-CSF concentration was increased in COPD, independent of disease severity, and in moderate to severe asthma. Increased bronchial submucosal expression of both GM-CSF and its receptor was a particular feature of severe asthma. Our findings therefore do support a potential role for GM-CSF in asthma and possibly COPD. Efficacy studies of therapeutic strategies targeted at GM-CSF are eagerly awaited and will further define the functional importance of GM-CSF in airways disease.
